# A kinetic-based stopped-flow DPPH^•^ method

**DOI:** 10.1038/s41598-023-34382-7

**Published:** 2023-05-10

**Authors:** Lucrezia Angeli, Ksenia Morozova, Matteo Scampicchio

**Affiliations:** Faculty for Agricultural, Environmental and Food Sciences, Free University of Bolzano, Piazza Università 1, 39100 Bolzano, BZ Italy

**Keywords:** Spectrophotometry, Small molecules

## Abstract

The reaction kinetics of antioxidants with free radicals is crucial to screen their functionality. However, studying antioxidant-radical interactions is very challenging for fast electron-donor substances, such as ascorbic acid, because the reaction ends in a few seconds. Accordingly, this work proposes a rapid and sensitive method for the determination of the absolute rate constant of the reaction between fast antioxidants and DPPH^•^. The method consists of a stopped-flow spectrophotometric system, which monitors the decay of DPPH^•^ during its interaction with antioxidants. A kinetic-based reaction mechanism fits the experimental data. Kinetic parameters include a second order kinetics (*k*_*1*_) and, depending on the type of antioxidant, a side reaction (*k*_*2*_). Ascorbic acid was the fastest antioxidant (*k*_*1*_ = 21,100 ± 570 M^−1^ s^−1^) in comparison with other eleven phenols, showing *k*_*1*_ values from 45 to 3070 M^−1^ s^−1^. Compounds like catechin, epicatechin, quercetin, rutin, and tannic, ellagic and syringic acids presented a side reaction (*k*_*2*_ from 15 to 60 M^−1^ s^−1^). Among seven fruit juices, strawberry was the fastest, while red plum the slowest. Overall, the proposed kinetic-based DPPH^•^ method is simple, rapid, and suitable for studying the activity and capacity of different molecules, and food samples rich in fast antioxidants, like fruit juices.

## Introduction

The reaction between phenolics and DPPH^•^ (2,2-diphenyl-1- picrylhydrazyl) radical is one of the most common assays that is used to determine the total antioxidant capacity of foods^1^. This mechanism is often used as model for the reactions of peroxyl radicals with phenolic compounds. Its working principle is based on the reaction between 2-2-diphenyl-1-pycrylhydrazyl (DPPH^•^) and antioxidants. DPPH^•^ absorbs light at 515 nm (purple). In protic solvents, like methanol, there is an electron transfer from the antioxidant to the DPPH^•^, and the resulting DPPH-radical becomes yellow. The changes in the absorption observed before and after the addition of antioxidants, during a fixed time (i.e., 30–60 min), are generally used to express the total antioxidant capacity (TAC). The total antioxidant capacity is generated by the complex chemical composition as well as by the synergistic effect generated by the multitude of active principles existing in the fruit, for example polyphenols, carotenoids, proteins, vitamins, amino acids, lipids. This index is influenced by the amount of antioxidants that reacted with a known amount of DPPH radicals and their interaction in a natural extract^2,3^.

Recently, the classical DPPH^•^ assay has been turned into a more comprehensive kinetic-based DPPH^•^ method, which allowed not only to express the TAC value of individual antioxidants and food extracts, but also to determine the absolute rate constants and the stoichiometry of the reaction^4–6^. This reaction was described by a two-steps mechanism, as shown in Eqs. (1–2)^4,7^:

1 $${\text{AH}} + n^{ \cdot } {\text{DPPH}}^{ \cdot } \to ^{{k_{1} }} {\text{A}} \cdot + {\text{DPPH}} - {\text{H}}$$

2 $${\text{A}}^{ \cdot } + {\text{DPPH}}^{ \cdot } \to ^{{k_{2} }} {\text{Products}}$$

where AH is a general antioxidant, *n* is the stoichiometric factor, A^•^ is the antioxidant radical, and DPPH-H is the reduced form of the DPPH^•^ radical. The simulation and fitting of the transient changes of DPPH^•^ allowed to express (a) the antioxidant activity in terms of absolute rate constants (Eq. (1)), (b) the antioxidant capacity in terms of the stoichiometry factor (the *n* value), and, finally, (c) to reveal the presence of side reactions (Eq. (2)).^7^

However, kinetic methods are difficult to be applied for studying fast antioxidants, like ascorbic acid. Ascorbic acid is a water-soluble vitamin, that is essential for human health, i.e., to treat scurvy, repair tissues and regulate the immune system^8–10^. Many edible plants, like fruits, vegetables, and their juices, are important dietary sources of ascorbic acid^11^. Vitamin C is also widely used as additive in foods to slow product deterioration, like enzymatic browning^12^. Despite its widespread use, the radical scavenging activity of ascorbic acid is not well characterized, likely, because its reaction with radicals, is so fast that the reaction goes to completion within a few seconds. This makes difficult to perform kinetic studies with spectroscopic measurements, i.e., performed with classical glass cuvettes. In such cases, the reduced form of ascorbic acid falls rapidly to zero even during its mixing with radicals, like DPPH^•^^13^.

To overcome such limitations, the kinetic-based DPPH^•^ method could be greatly improved if implemented with a stopped-flow technique. A stopped flow apparatus is especially suitable for studying fast reactions^14–16^, in which two or more reactants can be rapidly mixed, and delivered through a flow cell detector with a dead time of just a few microseconds. Accordingly, this work was aimed at developing a kinetic-based stopped-flow DPPH^•^ method for the determination of the activity and capacity of fast antioxidants, like ascorbic acid, and food samples naturally rich in ascorbic acid, like fruits juices. Overall, the results will contribute to improve the understanding of the functional behaviour of fast antioxidants.

## Materials and methods

### Chemicals

Methanol, DPPH^•^, Folin reagent, Na_2_CO_3_, and standards reagents (ascorbic acid, α-tocopherol, Trolox, catechin, epicatechin, quercetin, rutin, tannic, ellagic, 3,4-dihydroxybenzoic, and syringic acid) were all purchased from Sigma-Aldrich (St. Louis, MO, USA) at the highest available grade. Phloretin with a purity higher than 98% was purchased from Tokyo Chemical Industry (Zwijndrecht, Belgium). Stock solutions of each antioxidant were prepared in methanol at a final concentration of 10 mM. Stock solutions of DPPH^•^ were prepared in methanol at the concentration of 2.5 mM. All solutions were prepared daily.

### Food samples

Fruit samples (apple, apricot, grapefruit, kiwi, peach, red plum and strawberry) were purchased from a local market. The juice was extracted from of 1 kg of ripen fruits by a kitchen extractor (Kenwood, Italy). An aliquot of the extract (30 mL) was centrifugated at 10,000 rpm at 10 °C (SL 16 R centrifuge, Thermo Scientific, Waltham, MA, USA) and the supernatant was filtered (qualitative filter paper, 301, 12–15 μm, VWR, Leuven, Germany). The resulting juice sample was weighted, labelled, and stored at −80 °C.

### Total phenolic content

Total phenolic content (TPC) was estimated with the Folin–Ciocalteu’s reagent using the method of Singleton et al. with slight modifications^17^. Briefly, a volume of the juice sample (130 μL) was mixed with distilled water (1 mL) and the Folin reagent (130 μL). After 5 min, 130 μL of Na_2_CO_3_ solution (20%) were added. The mixture was vortexed, incubated for 2 h in the dark at 25 °C and transferred in a microplate well (UV-Star® microplate, 96 wells, Greiner Bio one, Frickenhausen, Germany). The absorbance was read at 765 nm with the spectrophotometer (Infinite M Nano^+^ , Tecan, Männedorf, Switzerland). Results were expressed as mg/100 mL of gallic acid equivalents (GAE) from a calibration curve built with standard solutions of gallic acid (R^2^ = 0.996).

### Classical DPPH^•^ assay

The classical DPPH^•^ assay was performed with the method of Ding et al. with slight modifications^18^. A methanolic 2.5 mM stock solution of DPPH^•^ was prepared and diluted to 200 μM. Then, 100 μL of the working solution and 100 μL of sample were added to a 96-wells plate and the absorbance was read after 1 h in the dark at 515 nm with a spectrophotometer. The concentration of the fruit juices was standardized at 60 μM of GAE, to make the results comparable with those of the kinetic approach (see below). The analysis was repeated in triplicates and results were expressed as % inhibition of DPPH^•^, using Eq. (3):

3 $$\% Inhibition = \frac{{t_{0} - t_{60} }}{{t_{0} }}*100$$

where t_0_ and t_60_ correspond to the sample absorbance of DPPH^•^ observed at 0 and 60 min, respectively.

### Stopped-flow kinetic-based DPPH^•^ method

The kinetic-based DPPH^•^ method was performed with a stopped-flow system (RX2000, Applied Photophysics, Leatherhead, UK) equipped with a pneumatic pump, a quartz flow-cell and a Cary 60 UV–VIS spectrophotometer (Agilent Technology, Santa Clara, CA, USA). The stopped-flow system had two syringes, one loaded with 200 μM DPPH^•^ solution, and the other with the antioxidant at concentrations between 20 and 200 μM. It should be noted that the DPPH^•^ and antioxidant solutions were prepared at a doubled concentration than the one desired, since there is a 1:1 dilution after the mixing of the two reagents. The concentration of the fruit juice samples was therefore standardized at 60 μM of GAE. Priming was performed before every run by flowing the two reagents in the system. As soon as the pneumatic drive was pressed, equal volumes of the two solutions were mixed and transferred into the quartz flow cell, with a max delay of 6 ms. The resulting absorbance of the reaction mixture was recorded every 18 ms, at a wavelength of 515 nm, by the UV spectrophotometer. The concentration of the DPPH^•^ was calculated from the recorded absorbance signal by applying the Beer–Lambert law. At this purpose, the molar extinction coefficient of DPPH^•^ in methanol (ɛ_515_) was determined from the absorbance of increasing standard solutions, leading to values of ɛ_515_ equal to 11,200 ± 400 M^−1^ cm^−1^, in agreement with that found elsewhere^19^.

Simulation and fitting of the reaction kinetic data were performed with the software Copasi (version 4.29). Simulated od DPPH^•^ consumption were obtained from solving a set of differential equations derived by the law of mass action applied to Eqs. (1–2). Optimal values of the kinetic parameters (*k*_*1*_ and *k*_*2*_) and the reaction stoichiometry (*n*) were obtained by minimizing, through iteration, the sum of squared errors between the experimental and simulated data. In details, the stoichiometric factor was derived from the ratio between the optimal concentration found by the software Copasi and the known concentration added in the reaction mixture^20^. A detailed description of the steps needed to simulate and run the Copasi software are described as “Supplementary Material”. Each experimental point reported in the manuscript is the average of three independent replicates performed in different days with fresh solutions and reagents.

### Statistics

Statistical analysis of kinetic data was performed with Copasi software (version 4.29). Basic statistics, such as mean and standard deviation, were obtained by Microsoft Excel (Version 2211 Build 16.0.15831.20098). For Pearson correlation tests, OriginPro was used (OriginPro 2023, learning edition).

## Results and discussion

### Application on antioxidant standards

**Figure 1:**
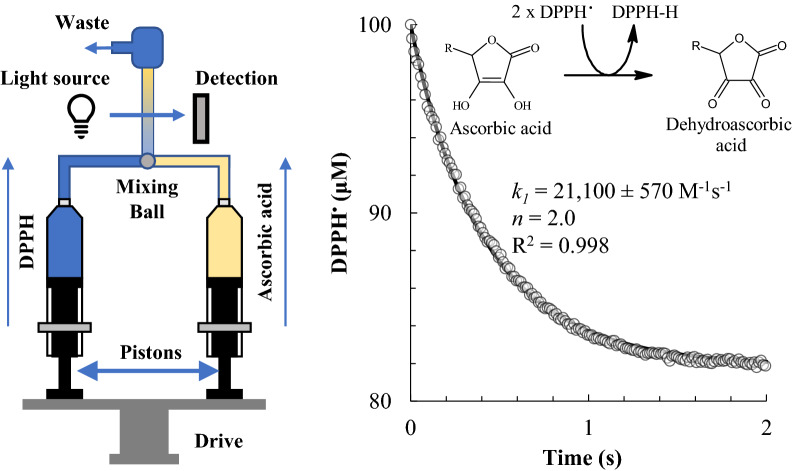
(Left) A stopped-flow system consisting of two syringes with pistons that push the two reagents (e.g. DPPH^•^ and ascorbic acid) into a mixing chamber and then in the quartz flow cell, driven simultaneously by the same driving motor. (Right) Kinetic curve and fitting of the reaction between 10 μM vitamin C and 100 μM DPPH^•^ with the reaction mechanism and the kinetic parameters.

Figure 1 (right) shows the changes in the DPPH^•^ concentration during its reaction with vitamin C. The reaction was so fast that an experiment had to be set up with a stopped-flow technique to record enough absorption signals. Stopped flow is an experimental technique for studying rapid chemical reactions with a half-time of the order of milliseconds^21^. The setup here consisted in two syringes filled, respectively, with 100 µM of DPPH^•^ and 10 µM of ascorbic acid (Fig. 1 left). Equal volumes of the two solutions were rapidly mixed and driven into a high-efficiency mixer and transferred into a quartz flow cell, with an overall dead time of only 8 ms. The reaction was next monitored by recording the maximum absorbance signal at 515 nm every 18 ms. This assured a sufficient number of data points that were necessary to robustly quantify the kinetics. It should be noted such experiments performed without a stopped-flow apparatus, i.e., with a classical quartz cuvette, are inaccurate because they do not allow the measurement of the initial part of the DPPH^•^ decay curve, which is generally lost during the time required for mixing the reagents.

After having collected enough experimental points, the rate constants of the reaction between DPPH^•^ and ascorbic acid were obtained by numerical methods. Briefly, a system of ordinary differential equations (ODEs) – one for each chemical species taking parts to the mechanism of Eqs. (1–2) – was derived by using the law of mass action. This states that the rate at which a chemical species is generated or consumed is equal to the product of the concentrations of the reactants times a rate constant (*k*_*x*_). Next, the resulting set of ODEs were numerically solved with the LSODA algorithm^22^ of the ODEPACK library^23^, by setting the initial “known” concentrations of ascorbic acid and DPPH^•^. LSODA algorithm was chosen because it can handle stiff differential equations, i.e., when fast and slow reactions occur simultaneously. Next, simulations of the transient changes of DPPH^•^ were varied by changing the *k*_*1*_ and *k*_*2*_ values, until the distance to the observed time-courses was minimized. The entire iterative procedure was managed automatically by Copasi software. A detailed tutorial about this procedure is reported as “Supplementary Material” (see text in SI).

This approach led to a rate constant for ascorbic acid equal to *k*_*1*_ = 21,100 ± 570 M^−1^ s^−1^ (R^2^ = 0.998). Such high value is typical for fast interactions with the DPPH radicals. No significant contribution of the side reaction (*k*_*2*_) was observed. This was mainly because ascorbyl radicals produced in Eq. (1) oxidized to dehydroascorbic acid and later underwent irreversible degradation processes^24^. The stoichiometry of the reaction was 2.0. This was obtained as the ratio between the observed changes in DPPH^•^ concentration and the initial concentration of ascorbic acid (*n* = ∆[DPPH^•^] / [AH]_0_). For instance, Fig. 1 (right) shows that when 10 µM of ascorbic acid were used, 20 µM of DPPH^•^ were consumed, leading a stochiometric factor *n* = 2.0^25,26^.

**Figure 2:**
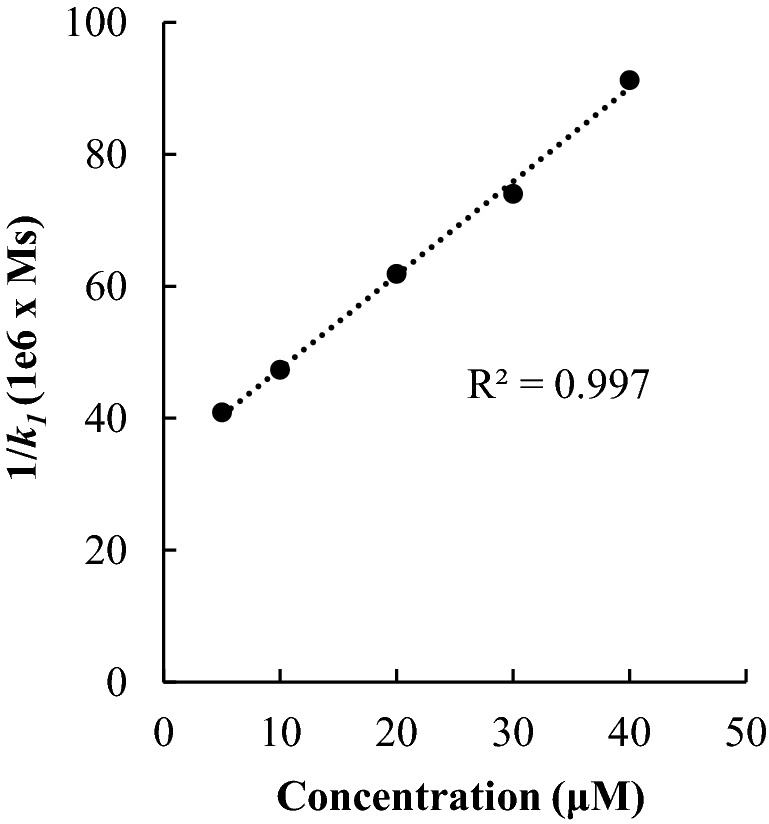
Rate constant values 1/*k*_*1*_ as a function of the concentration of ascorbic acid.

Figure 2 shows the rate constant values obtained for different initial concentrations of ascorbic acid. For higher concentrations, the rate constants were lower. Indeed, a plot of 1/*k*_*1*_ against the concentration showed a linear trend (R^2^ = 0.997). Variations in the rate constant as a function of the reactant concentration have been also previously reported with antioxidants having carboxylic functionalities^4,7^. A possible explanation is because for high concentrations of antioxidants, the formation of phenolic anions results inhibited (which is the reactive species), slowing down the overall electron transfer process^19^. This effect of the concentration on the resulting value of the rate constant highlights the importance of indicating the concentration of antioxidant used in the DPPH^•^ method. Without such an indication, the DPPH^•^ method, whether in its classical or kinetic version, is irreproducible.

**Figure 3:**
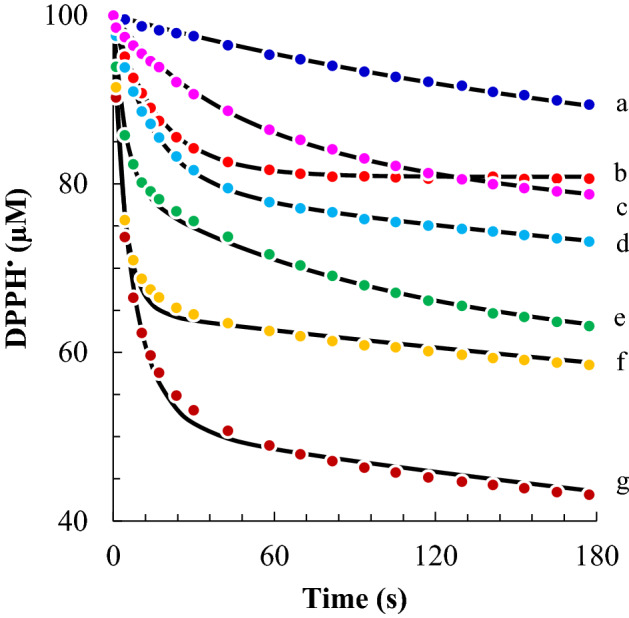
Kinetic curves with fitting of the reaction between 50 µM of phloretin (**a**), 10 µM of alpha-tocopherol (**b**), catechin (**c**), epicatechin (**d**), quercetin (**e**), ellagic acid (**f**), 2 µM of tannic acid (**g**) and 100 μM of DPPH^•^.

The utility of the stopped-flow kinetic method was next extended also to other antioxidants. Figure 3 shows the transient signal of DPPH^•^ (100 µM) after the rapid mixing with 50 µM of (a) phloretin and equimolar concentrations (10 µM) of, (b) α-tocopherol, (c) catechin, (d) epicatechin, (e) quercetin, (f) ellagic acid, and a diluted solution of (g) tannic acid (2 µM). These antioxidants were chosen because they represent different classes of antioxidants of food interest (i.e., hydrophilic vitamins, lipophylic vitamins, polyphenols). Table 1 sums up their kinetic parameters, also in comparison with % of inhibition obtained with the classical DPPH^•^ assay. All compounds were much slower antioxidants than ascorbic acid as their rate constants were from 10 to 500 times lower than that of ascorbic acid.

**Table 1:** Values for the % of inhibition and stoichiometry obtained from the classical DPPH^•^ assay and rate constant of the main reaction (*k*_*1*_), the side reaction (*k*_*2*_), the stoichiometric factor (*n*), and the correlation (R^2^) obtained from the stopped-flow kinetic method.

Sample name	Classical DPPH^•^		Kinetic-based DPPH^•^ assay			
	% Inhibition	*n*	*k*_*1*_ (M^−1^ s^−1^)	*k*_*2*_ (M^−1^ s^−1^)	*n*	R^2^
Ascorbic acid	18.8 ± 0.1	1.9	21,100 ± 570		2.0	0.998
Rutin	48.3 ± 0.3	4.8	3070 ± 300	60.0 ± 4.3	1.8	0.994
Ellagic acid	64.6 ± 0.4	6.5	2900 ± 160	18.7 ± 1.4	3.7	0.991
Quercetin	48.6 ± 0.3	4.9	2380 ± 81	52.5 ± 1.3	2.0	0.997
Tannic acid	72.6 ± 0.6	36	1730 ± 64	21.8 ± 1.8	24	0.992
Epicatechin	57.6 ± 0.4	5.8	778 ± 43	30.3 ± 1.8	2.1	0.999
Trolox	19.6 ± 0.2	2.0	657 ± 47		2.1	0.999
α-tocopherol	18.5 ± 0.1	1.8	601 ± 11		2.1	0.999
Syringic acid	26.7 ± 0.2	2.7	401 ± 24	14.9 ± 1.3	2.0	0.999
Catechin	48.6 ± 0.4	4.9	252 ± 5	25.8 ± 1.1	1.7	0.999
Protocatechuic acid	25.2 ± 0.2	2.5	172 ± 13		2.1	0.998
Phloretin	40.5 ± 0.4	0.8	45.1 ± 2.6		0.4	0.998

The high reactivity of ascorbic acid in comparison with the other listed in Table 1 would not have been predicted based on the classical DPPH^•^ assay^27,28^. Surprisingly, the results of the classical assay showed that ascorbic acid was one of the antioxidants least able to inhibit DPPH^•^ oxidation. However, this apparent contradiction is expected considering that the kinetic-based DPPH^•^ assay highlights the velocity of the reaction (i.e., the antioxidant activity), whereas, the classical DPPH^•^ expresses only the amount of antioxidants that have reacted (i.e., the antioxidant capacity)^19^. Based on these findings, we agree with the recent criticisms raised around the abuse of the classical DPPH^•^ assay in so many research studies for ranking antioxidants and natural extracts^19,27^. Instead, the kinetic-based DPPH^•^ method offers several advantages.

One advantage is exemplified here with tannic acid. This commercial molecule is composed of several gallic acid moieties, resulting in 24 active hydroxyl groups. Accordingly, when 2 μM of tannic acid were added in the reaction mixture, the fitting program led to *k*_*1*_ and *k*_*2*_ respectively of 1,730 ± 65 M^−1^ s^−1^ and 21.8 ± 1.8 M^−1^ s^−1^, and a stoichiometric factor of 24. Contrarily to the classical DPPH^•^ assay, the kinetic-based DPPH^•^ method provides a stoichiometric value that matches the number of hydroxyl groups in the molecule^29^. Also, we could conclude that tannic acid is an antioxidant with medium activity (based on *k*_*1*_), but very high capacity (based on *n* value).

The next example is phloretin, a dihydrochalcone composed of two aromatic rings with hydroxyl groups in positions 2, 4, 6 and 4′ connected by a three-carbon carbonyl. Based on the main rate constant (*k*_*1*_ = 45.1 ± 2.6 M^−1^ s^−1^), phloretin showed lower antioxidant activity compared to other studied compounds. In addition, when 50 μM of phloretin were added in the reaction mixture, just 2 µM of DPPH^•^ were consumed, resulting in a stoichiometry factor of 0.4. Although some authors consider phloretin as a potent antioxidant towards lipid peroxidation, it has also been demonstrated that the H-atom transfer mechanism is preferred than the electron-transfer^8^, which is involved in the DPPH^•^ reaction^30^. This can explain phloretin showed lower antioxidant activity and capacity in the stopped flow DPPH^•^ assay.

The mechanism of the proposed kinetic-based DPPH^•^ method takes into account side reactions (see Eq. (2)), which explains its further utility. Several antioxidants like catechin, epicatechin, quercetin, rutin, tannic, ellagic and syringic acids in fact exhibited a side reaction (*k*_*2*_). This is related to the interaction between antioxidant radicals and DPPH• radicals^7^, leading to new stable, non-radical complex, which slows down the DPPH^•^ consumption. Foti reported that semiquinone radicals, that were produced by the reaction between DPPH^•^ and ubiquinone, lead to several possible quenching process, one being their further oxidation to ubiquinone and the regeneration of DPPH-H^31^. Although the envisaging among the possible quenching processes of all phenolics is outside the scope of this work, however, it becomes evident that the contribution of a side reaction must be considered. From one side, the presence of a side reaction means that the DPPH^•^ method is not a simple kinetic process. Therefore, the reaction kinetic behind the DPPH^•^ method studied with pseudo-first order conditions, i.e., with [AH]_0_ >  > [DPPH^•^]_0_ might be an oversimplification.

On the other hand, the side reaction affects the resulting kinetic values of Eq. (1), as well as the reaction stoichiometry. This also implies that any kinetic result obtained using either pseudo-first order or elementary second orders rate laws^29,32,33^, i.e., if obtained without considering side reactions, should be deemed with caution because they may oversimplify the reactivity of phenols.

Finally, the correlation between the stoichiometry values determined with the classic *vs* kinetic-based DPPH^•^ method is weak (R^2^ = 0.39, see SI). This is because the classical DPPH^•^ assay is measured at a fixed moment (i.e., after 60 min of reaction), assuming that the reaction is finished at this point. Thus, the overall stoichiometry includes both the initial concentration of the antioxidant and also the scavenging of further DPPH^•^ radicals related to side reactions, that causes an overestimation of the stoichiometry^27^. Instead, since the kinetic-based DPPH^•^ method accounts for the possible contribution of even slow side reactions, the resulting stoichiometry is calculated from the initial concentration of the antioxidant^28^.

### Application on food samples

The utility of the stopped flow kinetic-based DPPH^•^ method was finally extended to complex food matrices. Table 2 reports the results of both kinetic-based and classical DPPH^•^ assay for seven fruit extracts, together with the total phenolic content (TPC) of the extracts. The latter measurement was needed because fruit extracts consist of mixture of different antioxidant compounds that can contribute to the total reactivity in different ways. Therefore, the measurement of the TPC was useful to estimate the initial antioxidant concentration [AH]_0_.

**Table 2:** Values for the TPC (GAE), rate constant of the main reaction (*k*_*1*_), the side reaction (*k*_*2*_), *n* obtained with the kinetic approach, the correlation (R^2^), and the % of inhibition and *n* obtained with the classical method.

Sample name	TPC	Kinetic-Based DPPH^•^ Method				Classical DPPH^•^	
	GAE (mg/100 mL)	*k*_*1*_ (M^−1^ s^−1^)	*k*_*2*_ (M^−1^ s^−1^)	*n*	R^2^	% inh	*n*
Apple	20.1 ± 0.98	161 ± 16	–	0.3	0.99	17.1 ± 0.85	0.6
Apricot	13 ± 0.65	297 ± 19	–	0.2	0.99	18.5 ± 0.92	0.6
Grapefruit	26.7 ± 1.3	2260 ± 59	–	3.2	0.99	95.7 ± 4.8	3.2
Kiwi	41.3 ± 2.1	3550 ± 80	–	3.2	0.99	95.9 ± 4.4	3.2
Peach	9.6 ± 0.5	694.0 ± 6.3	–	0.7	0.995	49.0 ± 2.4	1.6
Red plum	58.7 ± 2.9	134.0 ± 5.1	–	1.0	0.995	63.4 ± 3.2	2.1
Strawberry	61.3 ± 2.8	4880 ± 100	17.1 ± 0.21	1.9	0.997	79.9 ± 3.8	2.7

Based on TPC values, the results show that strawberry and red plum had the highest content in phenolic compounds, probably due to the high presence of anthocyanins, followed by the extracts from kiwi, grapefruits, and apples. Similar ranking was obtained by other authors^34–36^. Given the TPC values, the extracts were standardized to achieve a final equivalent concentration of 30 µM of GAE^37^.

**Figure 4:**
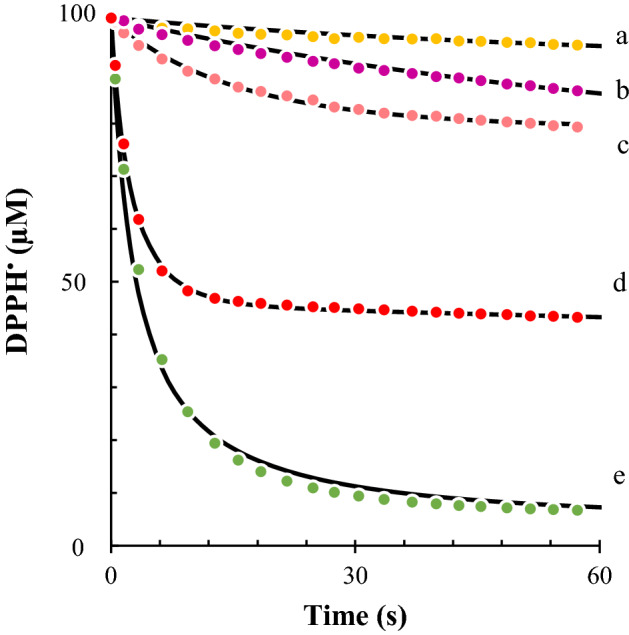
Kinetic curves and fittings of the reaction between 100 µM of DPPH^•^ and (**a**) apple, (**b**) red plum, (**c**) peach, (**d**) strawberry, and (**e**) kiwi extracts, standardized to 30 μM GAE. Other reaction conditions are in Fig. 1.

The resulting diluted extracts were next used in the kinetic-based DPPH^•^ method. Figure 4 shows the results of the assay together with the corresponding fitting curves. Thanks to the kinetic approach, a high number of kinetic points were recorded during the initial seconds of the reaction, allowing a robust fitting of the experimental time curves. The highest antioxidant activity was observed with strawberry extracts (i.e., the highest *k*_*1*_ value), followed by kiwi and grapefruit extracts. Again, the classical DPPH^•^ assay leaded to some opposite results. This is exemplified with red plum extracts, which showed the highest % of inhibition with classical DPPH^•^ approach, but one of the lowest antioxidant activities with the kinetic-based DPPH^•^ method. In summary, a kinetic-based DPPH^•^ method provides more complete information about the antioxidant activity and capacity of natural extracts.

## Conclusions

The kinetic-based stopped flow DPPH^•^ method allowed to determine the antioxidant activity and capacity of individual antioxidants and fruit extracts. The stopped flow kinetic method offers much more information compared to the classical DPPH^•^ assay, leading to absolute rate constant of the reaction, its stoichiometry value and providing evidence on the presence of side reactions. The stopped flow method is simple, rapid (a few seconds) and inexpensive. However, the kinetics observed depends on the wide variety of chemical constituents of fruit juices their concentration. Nevertheless, the kinetic based method extends the utility of the classical DPPH^•^ assay: while the latter can be useful to provide a general screening of antioxidant compounds, the kinetic approach allows to go in depth on the different characteristics of fast molecules. Thus, food industries can benefit from an extended version of the DPPH^•^ assay to focus on new antioxidants or on new fruit varieties with enhanced antioxidant activity. Future works should address their attention to elucidate the complex synergistic and antagonistic interactions between bioactive compounds to better estimate the activity and capacity of complex matrices.

## Supplementary Information

Supplementary Information.

## Data Availability

All the necessary data generated and/or analysed during the current study are included in this published article and its additional information, if needed, are available from the corresponding author on reasonable request.
