# Supplementary material for: A kinetic-based stopped-flow DPPH• method
Source: Sci Rep. 2023 May 10;13:7621. doi: 10.1038/s41598-023-34382-7 (PMC10172368; doi:10.1038/s41598-023-34382-7)
Supplement: Supplementary file 1 — Supplementary Information. [file 41598_2023_34382_MOESM1_ESM.docx]

# Supplementary Information

# A kinetic-based stopped-flow DPPH^•^ method

Lucrezia Angeli^1^, Ksenia Morozova^1^, Matteo Scampicchio^1*^

The use of Copasi to calculate the kinetic parameters of the reaction between 100 μM of DPPH^•^ and 10 μM of ascorbic acid is described in Figures s1-4.

**
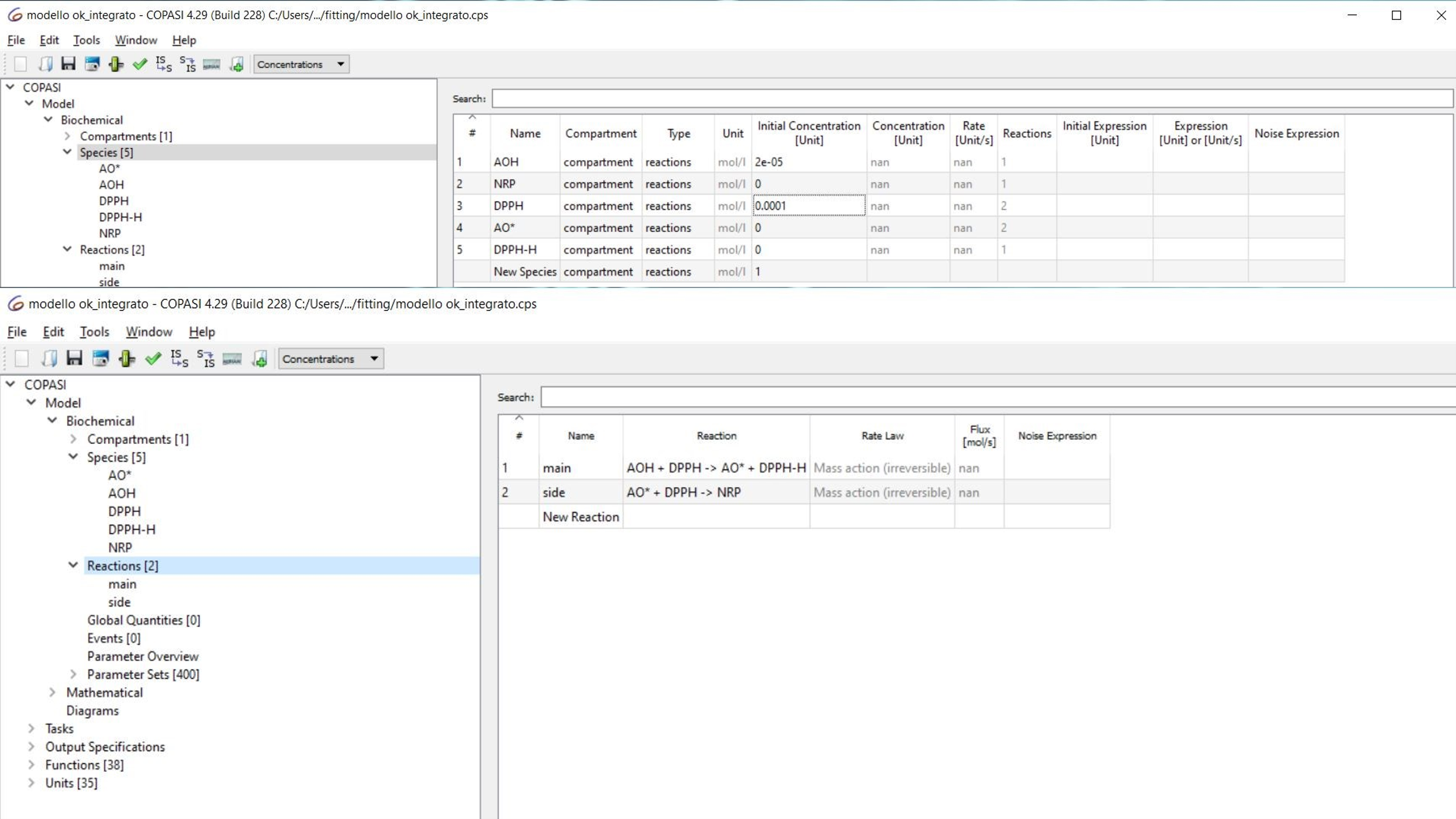
**

**Figure s1:** Model and reaction setup; it is important to set the correct initial concentration (mol/L) of DPPH^•^ of the single experiment.


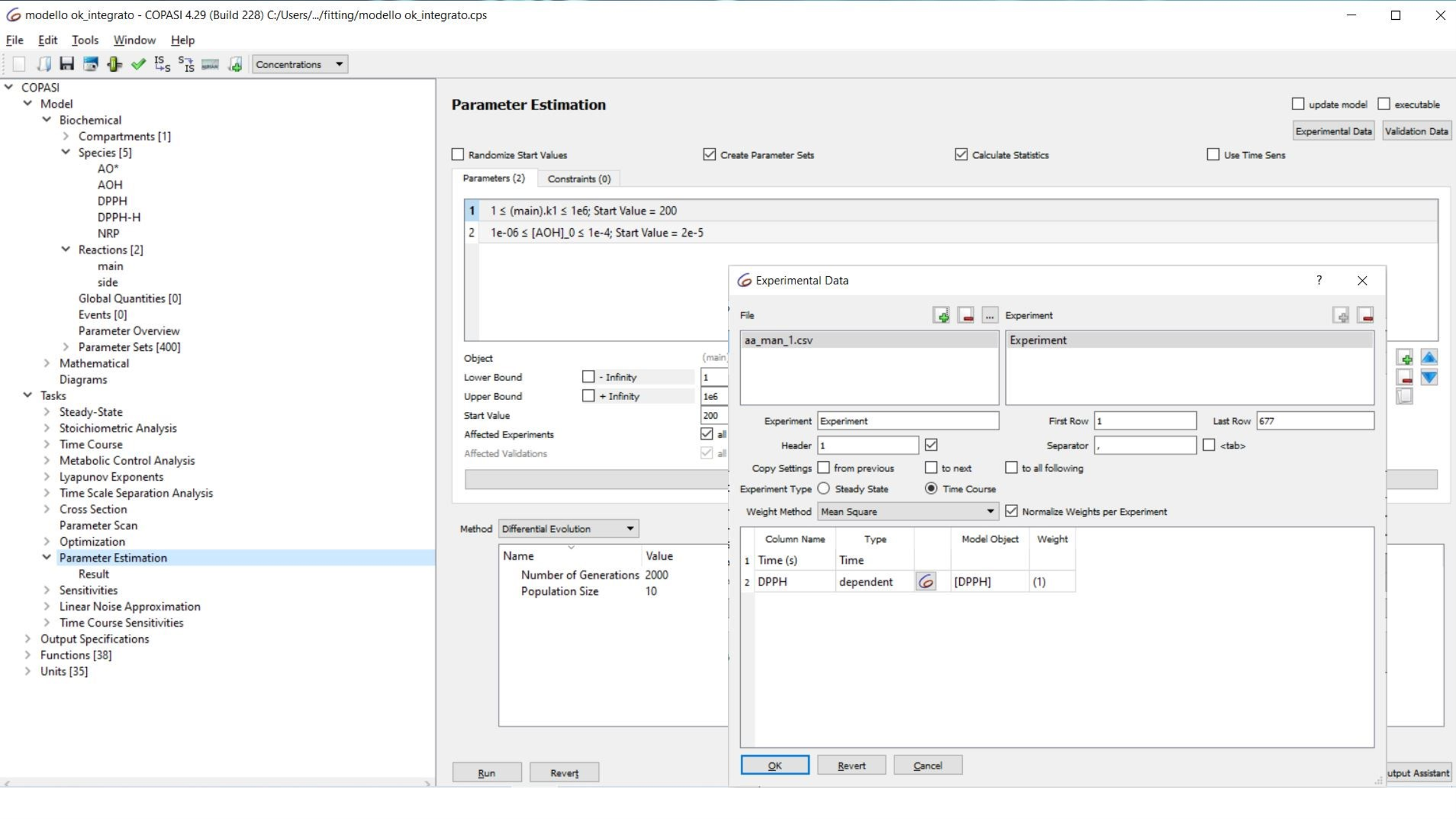


**Figure s2:** Setup for parameters (k_1_, k_2_, n) estimation. The side reaction can be added when needed.


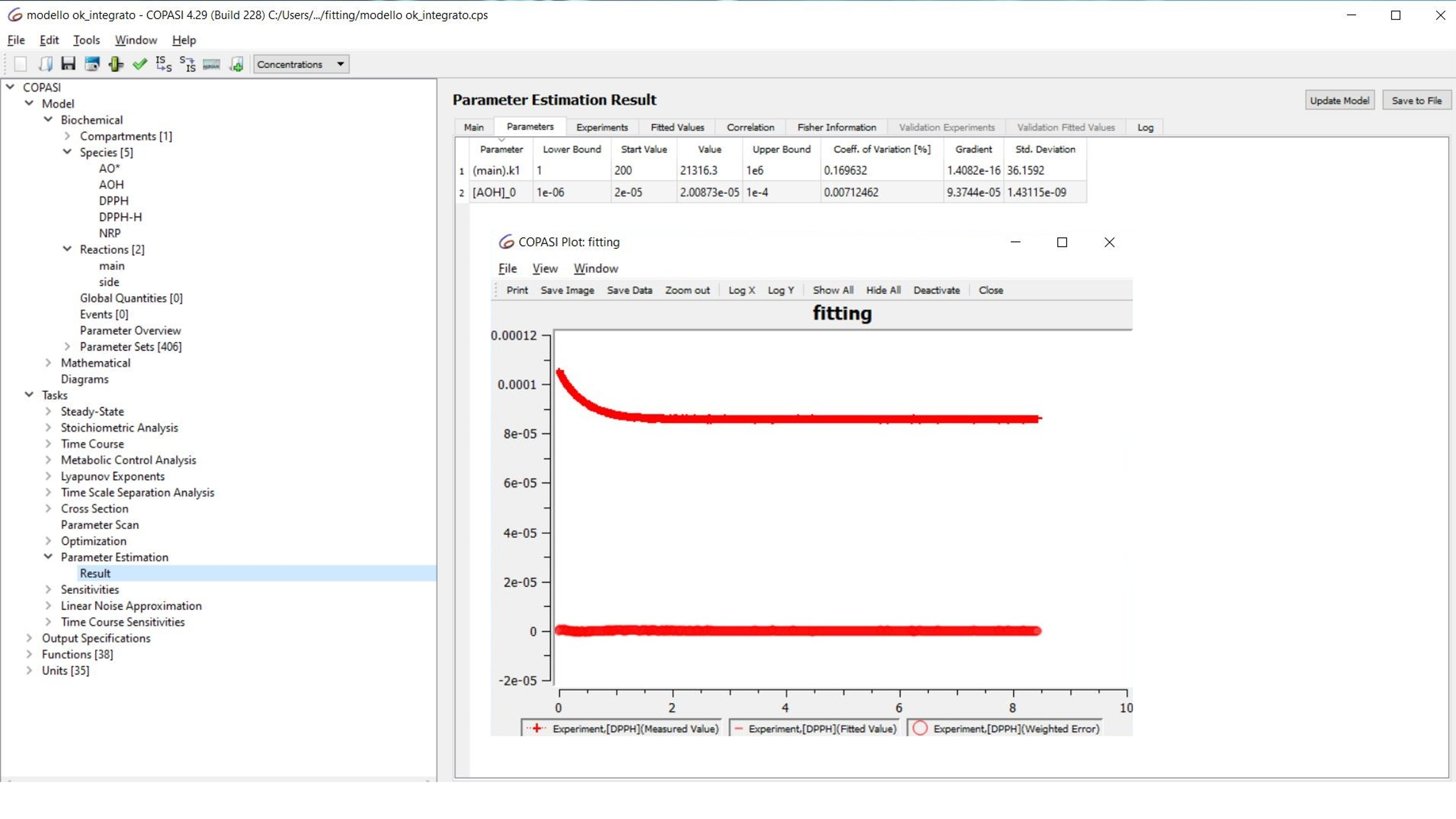


**Figure s3:** Fitting and parameter results of one replicate of the reaction between 100 μM of DPPH^•^ and 10 μM of ascorbic acid


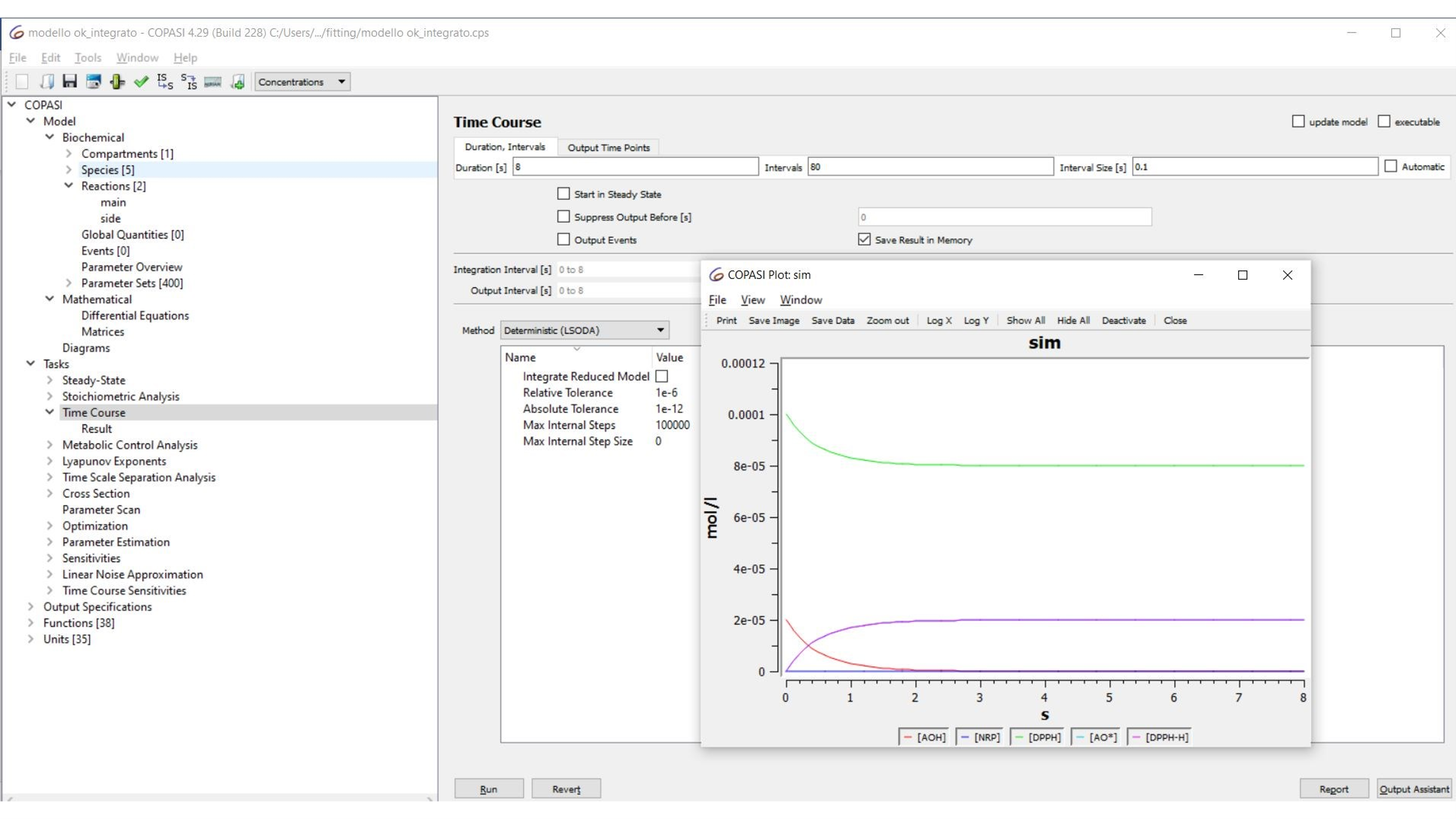


**Figure s4:** simulation of the reaction between 100 μM of DPPH^•^ and 10 μM of ascorbic acid


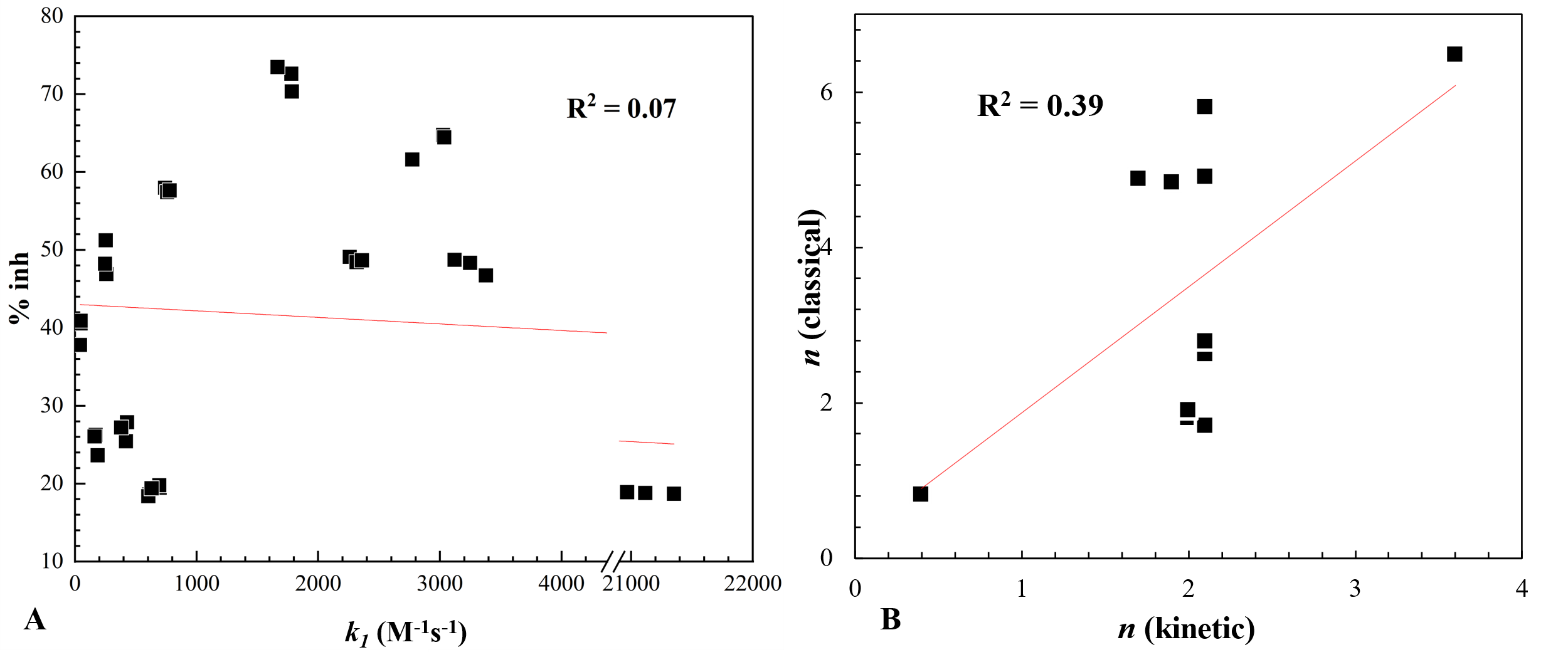


**Figure s5:** A - correlation between k_1_ values obtained from the kinetic DPPH^•^ assay and % of inhibition obtained from the classical approach; B – correlation between the stoichiometric value (n) obtained in the two methods.
